# The Overexpression of Scaffolding Protein NEDD9 Promotes Migration and Invasion in Cervical Cancer via Tyrosine Phosphorylated FAK and SRC

**DOI:** 10.1371/journal.pone.0074594

**Published:** 2013-09-18

**Authors:** Ni Sima, Xiaodong Cheng, Feng Ye, Ding Ma, Xing Xie, Weiguo Lü

**Affiliations:** 1 Department of Gynecologic Oncology, Women’s Reproductive Health Key Laboratory of Zhejiang Province, Women’s Hospital, School of Medicine, Zhejiang University, Hangzhou, Zhejiang, P. R. China; 2 Cancer Biology Research Center, Tongji Hospital, Tongji Medical College, Huazhong University of Science and Technology, Wuhan, Hubei, P. R. China; Ghent University, Belgium

## Abstract

NEDD9, a focal adhesion scaffolding protein, has been recently proposed to regulate invasion and metastasis in some cancer types, but unknown in cervical cancer. The aim of this study was to determine if NEDD9 was involved in the progression and metastasis of cervical cancer. The experimental results showed NEDD9 protein was overexpressed in cervical cancer compared with normal cervical epithelium tissues. Overexpression of NEDD9 was correlated with histological grading, lymph node metastasis, and FIGO stage of cervical cancer. Silencing NEDD9 resulted in tyrosine dephosphorylation of FAK and SRC oncoproteins, and decreased cell migration and invasion in the cervical carcinoma SiHa and HeLa cells. Overexpression of NEDD9 led to tyrosine phosphorylation of FAK and SRC oncoproteins, and increased cell migration and invasion. Moreover, tyrosine phosphorylation of NEDD9 was significantly decreased via suppressing tyrosine phosphorylation of FAK or SRC, suggesting a positive feedback loop of tyrosine phosphorylation between NEDD9 and FAK or SRC. In addition, our data showed that silencing NEDD9 decreased Vimentin expression and increased E-cadherin expression in cervical cancer cells, and vice versa. E-cadherin was subject to regulation of NEDD9, FAK and SRC, but altered neither tyrosine-phosphorylated nor total NEDD9. Our findings suggest that NEDD9 is overexpressed in cervical cancer tissues and cells, and overexpressed NEDD9 promotes migration and invasion in cervical carcinoma cells, probably via a positive feedback loop of tyrosine phosphorylation between NEDD9 and FAK or SRC.

## Introduction

Cervical cancer is the third most commonly diagnosed cancer in women worldwide, especially in developing countries. Annually, about 529,800 women encounter this disease, accounting for 9% of the total new cancer cases [1]. Although current treatment strategies such as radical hysterectomy and radiotherapy have good clinical outcomes, almost 275,100 deaths are attributed to cervical cancer each year. In addition, surgery is only suitable for early stage diseases and radiotherapy results in undesirable side effects, such as ovarian failure, vaginal stenosis, radiocystitis and radiation proctitis which influence the quality of the patient’s life [2]. Therefore, these facts underscore an urgent need for the development of more effective and novel therapeutic targets.

NEDD9 gene, first identified in neuronal precursor cells in 1992, was down-regulated during the development of mouse central nervous system [3]. Afterward, this gene was also found in other species and tissues. Law et al [4] screened a cDNA library for the genes that induced filamentous yeast budding of *S. cerevisiae* in order to find candidate genes that might coordinate cellular signaling and morphology. A “new” gene was identified and assigned as Human Enhancer of Filamentation 1 (HEF1). Another research team found that a novel 105 kD p130Cas-related protein was tyrosine phosphorylated by the engagement of β1 integrins in T lymphocytes and designated it as lymphocyte-type Crk-associated substrate (Cas-L) [5]. Thus, NEDD9 had two other independent names in history. NEDD9 proteins regulate protein complexes controlling cell cycle and apoptosis, migration, chemotaxis, and differentiation [6]. FAK and SRC were implicated as important targets of NEDD9 and they were phosphorylated and activated mutually, which was regarded as the “core regulation process” of NEDD9 [7].

In the past several years, altered expression of scaffolding protein NEDD9 has emerged as contributing to cancer metastasis in multiple cancer types, such as breast cancer [6,8], glioblastoma [9], melanoma [10], lung [11,12] and head and neck squamous cell carcinoma (HNSCC) [13]. However, no study to date is performed to determine if NEDD9 is associated with human cervical carcinoma. Recent reports have suggested that NEDD9 is overexpressed in some human carcinoma such as melanoma [10] and HNSCC [13]. Here we detected NEDD9 expression in human cervical cancer tissues and explored the role of NEDD9 in the progression of cervical cancer.

## Materials and Methods

### Patients and tissue sample collection

The samples of cervix and lymph node tissues and clinical parameters from 67 cervical cancer patients (38 cervical squamous carcinomas and 29 adenocarcinomas), who underwent surgery during 2005 to 2009 in Women’s Hospital, School of Medicine, Zhejiang University, were collected and detailed data were shown in Table S1. Moreover, 22 cervical normal tissue samples as controls were derived from patients who underwent hysterectomy because of benign gynecological diseases. No patients received chemotherapy or radiotherapy before the tissues were obtained. The study was reviewed and approved by the Ethical Committee of the Women's Hospital, School of Medicine, Zhejiang University. Written informed consent was obtained from all patients or their legal guardians.

### Immunohistochemistry

All tissues were immunohistochemical stained on 4-μm sections to assess NEDD9 expression in these samples. Mouse monoclonal antibody 2G9 against NEDD9 (Abcam, 1:1000) was employed as the primary antibody. EnVision^TM^ detection kit was used in the procedures and 3,3’-diaminobenzidine (DAB) was applied as chromogen. Each section was scored blindly for semi-quantitative determination of immunohistochemical staining intensity by a pathologist. Staining intensity was scored as follows: 0, no staining; 1, weak positivity; 2, moderate positivity; and 3, strong positivity. The composite immunohistochemistry scores were derived by summing the intensity scores multiplied by the cell proportion of this intensity score. For example, if the specimen showed moderate staining (scored 2) in 20% of the cells and strong staining (scored 3) in 30% of the cells, the composite immunohistochemistry scores would be (2 × 0.2) + (3 × 0.3) = 1.3. An average score was used for statistical analysis.

### Cell culture

Human cervical cancer cell lines SiHa (HPV16-positive), CaSki (HPV16-positive), HeLa (HPV18-positive), C33A (HPV-negative), human non-tumor keratinocyte line HaCaT and human embryonic kidney cell lines HEK293 and HEK293T were obtained from American Type Culture Collection (ATCC, Manassas, USA). The cells were cultured in Dulbecco’s modified Eagle’s medium (DMEM; GIBCO, USA) containing 10% fetal bovine serum (FBS) in a humidified atmosphere of 5% CO_2_ at 37°C.

### Immunofluorescence

Forty-eight hours after plating on glass coverslips, cells were fixed with 3.7% paraformaldehyde and incubated with mouse monoclonal antibody 2G9 (1:200) against NEDD9 as primary antibody. After rinse thrice with PBS, the cells were incubated with goat anti-mouse antibody conjugated to Alexa Fluor 647 (Invitrogen) diluted 1:1500 in 2% BSA as secondary antibody. The nuclei were marked with Hoechest33342 (Sigma).

### siRNAs Preparation and Transfection

Based on literature [14] and a free web-based tool (http://www.dharmacon.com), three pairs of siRNAs were designed against NEDD9 mRNA. siRNAs against HPV16 E6/E7 and E-cadherin were designed as described previously [15]. All of the siRNA duplexes (Table S2) were chemically synthesized by GeneChem Co., Ltd. (Shanghai, China). Transfections were performed in 6-well plates and using Lipofectamine™ 2000 Transfection Reagent (Invitrogen, USA) according to the manufacturer’s instructions.

### Lentiviral Production

The complete coding sequence of NEDD9 was amplified by PCR from the plasmids containing cDNA clone of NEDD9 (OriGene, USA) and inserted into a lentivirus gene transfer vector pLenti6/V5-DEST (Invitrogen, USA). The coding sequence of short hairpin RNAs (shRNAs) targeting green fluorescence protein (GFP) or NEDD9 (Table S2) were cloned immediately following a U6 pol III promoter into a lentivirus gene transfer vector (Invitrogen, USA). The vectors were subsequently packaged into lentiviral particles in HEK293T cells. The lentiviruses were used to infect cervical cancer cell as described previously [16]. Produced lentiviruses were titered and stored according to the manufacturer’s instruction.

### Quantitative PCR

Total RNAs were prepared from the treated cells using TRIzol reagent (Invitrogen, USA). Real time quantitative PCR (qPCR) was carried out using an 7300 Real-time PCR system (Applied Biosystems) and performed by SYBR Green dye according to the manufacturer’s instructions. Oligonucleotide primers were synthesized by Sangon Biotech Co., Ltd (Shanghai, China). as listed in Table S2. Relative quantification of the mRNA expression was calculated with the 2^−ΔΔCT^ method as described previously [17].

### Western blots and immunoprecipitation

Western blots were performed as described previously [18]. Cells were harvested and resuspended in lysis buffer for protein extraction. Fifty micrograms of total protein from each sample was subjected to a 10% SDS–PAGE gel electrophoresis. For immunoprecipitation, cells were disrupted in 250 μL of immunoprecipitation buffer. Equal amounts of protein (300 μg) were subjected to immunoprecipitation followed by Western blot analysis. Cumulative gray level of Western blot bands was obtained using ImageJ software (NIH, USA) for relative quantification quantitative analysis. Primary antibodies were specific for following proteins: NEDD9 (ab18056) from Abcam, FAK (3285), pFAK (3283), Src (2109), pSRC (2101), Phospho-Tyrosine (9411), Vimentin (3932) and E-cadherin (3195) form Cell Signaling, HPV16 E6 (MAB874), E7 (MAB8680) from Millipore, GAPDH (sc-59540) and β-actin (sc-1616-R) from Santa Cruz. Bands were visualized using an ECL Kit (Pierce, USA).

### Scratch wound-healing assay

Scratch wound-healing assay was performed to determine cell migration. Cells were infected with lentivirus, and 72 h later grown to full confluency. Then wounds (about 1 mm wide) were prepared using a micropipette tip scratched through the wells. The cell migration speed was calculated by measuring the distance migrated in 24 h. Photographs were taken using phase-contrast microscope (Olympus, Japan) on different time points after scratch.

### Transwell assays

In vitro cell invasion assays were carried out in Matrigel-based Transwell plates essentially as described previously by Pelletier et al [19] with slight modifications. 5 × 10^4^ Cells were plated into the Matrigel-coated upper chambers of the 24-well Transwell plates (Corning Costar, Cambridge, MA) with a pore size of 8 μm. The lower compartments were filled with medium supplemented with 20% fetal bovine serum. After 24 h of incubation, the non-migrated cells on the upper surface of the membranes were gently scraped away with cotton swabs and the migrated cells that had invaded to the lower surface were stained with crystal violet and counted. Cell migration assays were carried out in a similar way but without Matrigel.

### Statistical analysis

All experiments were repeated at least thrice. The data were analyzed with the software package SPSS 12.0. One way ANOVA and Student’s t-test were employed to compare the data. *p* Value less than 0.05 was considered significantly statistical.

## Results

### NEDD9 is overexpressed in human cervical cancer tissues and cell lines

**Figure 1:**
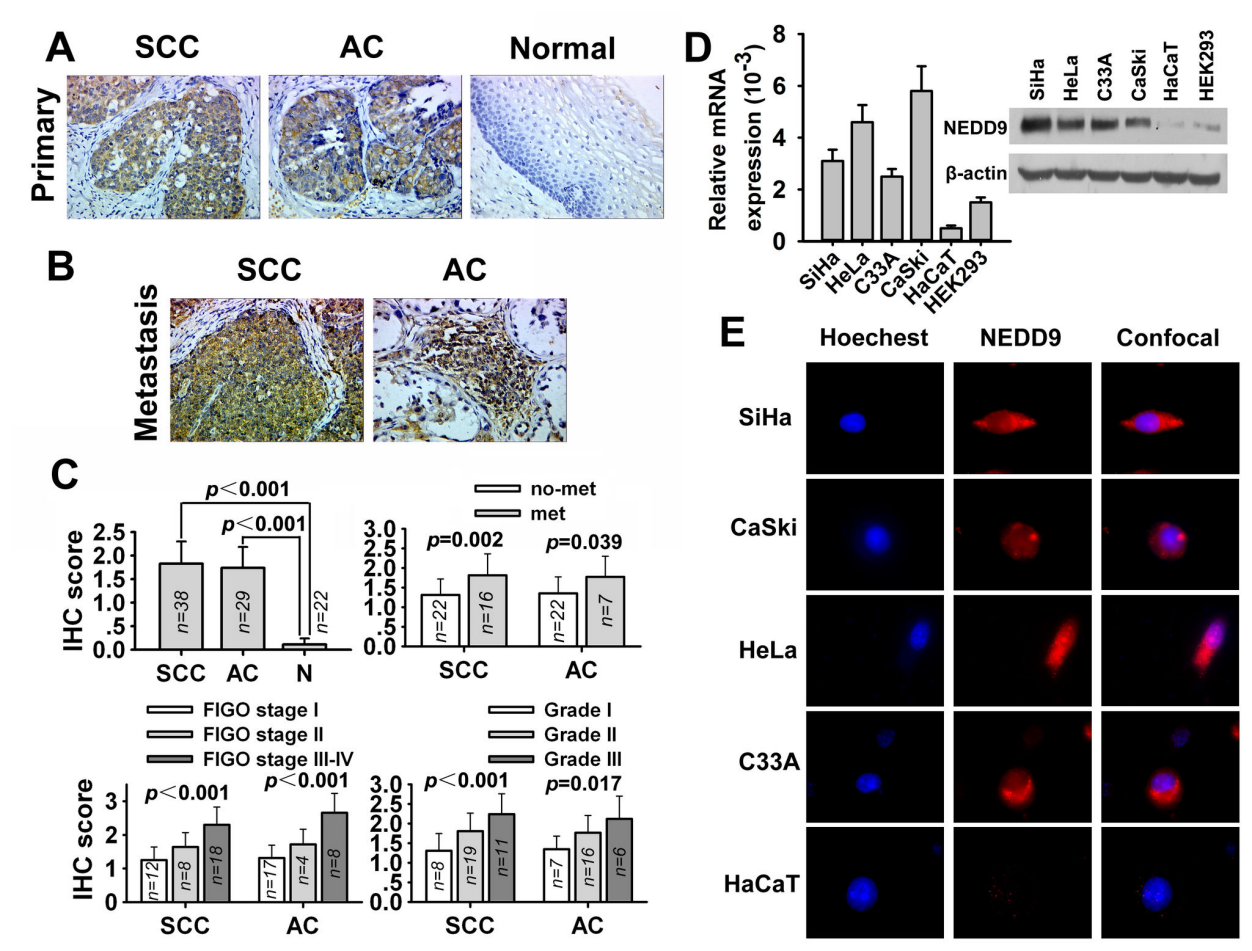
Expression of NEDD9 in human cervical cancer tissue and cells. (A) Immunohistochemical staining of NEDD9 in squamous carcinoma of the cervix (SCC), adenocarcinoma of the cervix (AC) and normal tissue of the cervix. Original magnification, ×400. (B) Immunohistochemical staining of NEDD9 in metastatic left external iliac lymph nodes of SCC and metastatic right obturator lymph nodes of AC. Original magnification, ×400. (C) Statistical analysis showed NEDD9 overexpression was correlated with FIGO stage, histological grading and metastasis. (D) Expression of NEDD9 mRNA and protein in several cell lines was assessed by quantitative PCR and Western blot analysis. (E) NEDD9 (red) localization and expression were assessed by immunofluorescent analysis in cervical carcinoma SiHa, CaSki, HeLa, C33A cells and human HaCaT keratinocytes. Cells were counter-stained with Hoechest33342 (blue) and visualized at 60× magnification.

To find a clue of the relationship between NEDD9 protein and human cervical carcinoma, we employed immunohistochemical analysis to determine the expression of NEDD9 protein in human cervical carcinoma tissues and analyzed the association of NEDD9 expression with human cervical carcinoma progression. We found that NEDD9 protein was overexpressed in most cervical carcinoma tissues but poorly expressed in normal cervical epithelium tissues (Figure 1A). In addition, NEDD9 protein was overexpressed in metastatic lymph nodes (Figure 1B). Moreover, NEDD9 overexpression was correlated with histological grade, metastasis, and FIGO stage (Figure 1C). Furthermore, as shown in Figure 1D, NEDD9 mRNA was overexpressed in four common cervical cancer cell lines (SiHa, HeLa, C33A and CaSki) regardless of HPV-positive or -negative but relatively poorly expressed in human keratinocyte line HaCaT and human embryonic kidney cell line HEK293. Western blot analysis showed that NEDD9 was highly expressed in the cervical cancer cell lines, similarly to those of qPCR. Immunofluorescence analysis showed that the protein of NEDD9 was overexpressed in cervical cancer cell lines and distributed mainly in cytoplasm (Figure 1E).

### Altered expression of NEDD9 influences migration, and invasion in cervical cancer cells

**Figure 2:**
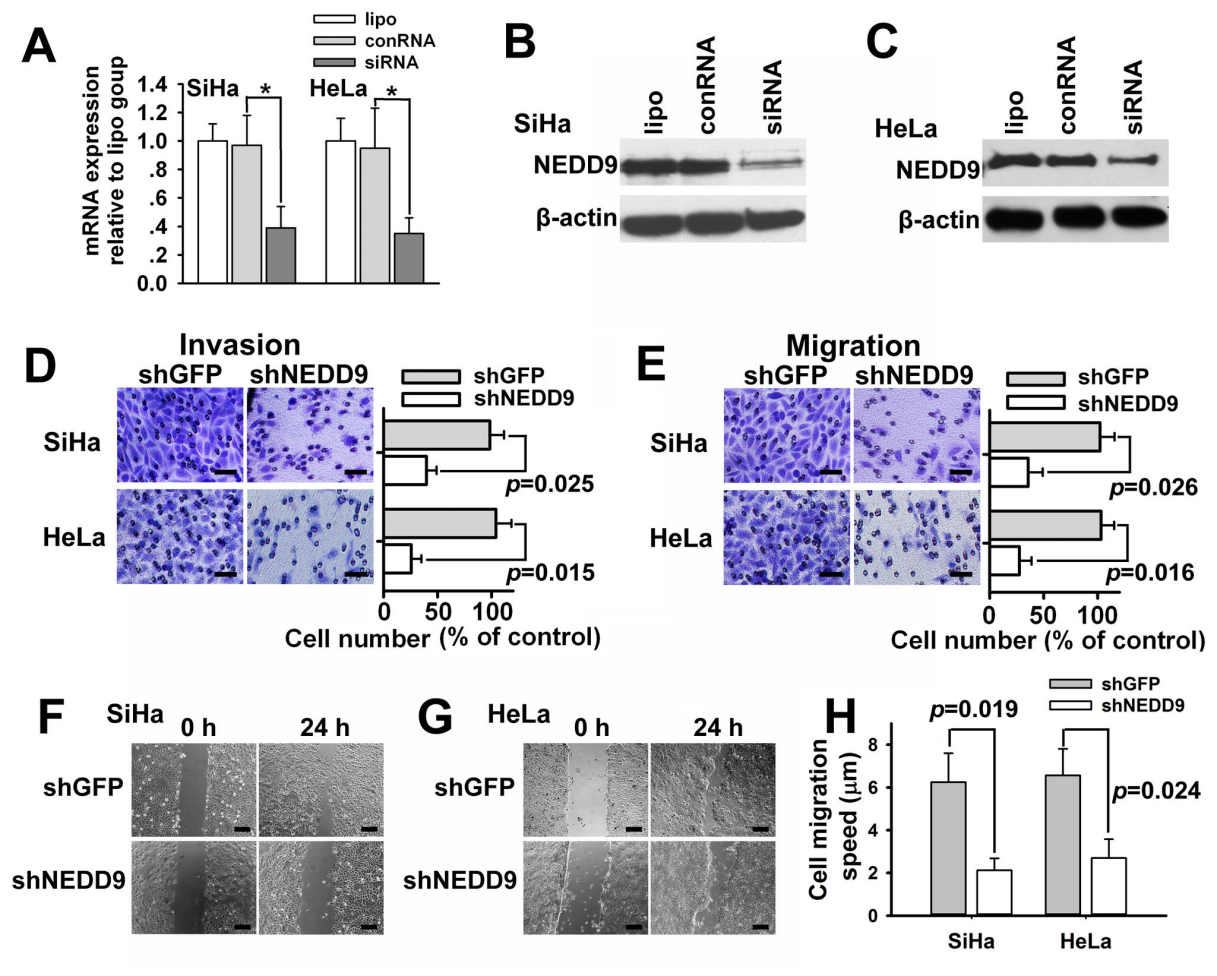
Silencing of NEDD9 resulted in reduced cell migration and invasion. NEDD9 was knocked down by siRNAs or shRNAs in cervical carcinoma SiHa and HeLa cells. (A) The interference effects were confirmed by quantitative PCR in SiHa and HeLa cells. Expression of NEDD9 was examined by Western blotting in SiHa (B) and HeLa cells (C). Cells were infected with lentiviral vectors encoding shRNA against NEDD9. The results of Transwell assay showed that lentiviral delivery of shRNA targeting NEDD9 resulted in reduced cell invasion (D) and migration (E) in SiHa and HeLa cells. The results of Scratch wound-healing assay further verified that silencing NEDD9 resulted in reduced cell migration (F, G and H). Scale bar, 100 μm. * *P* < 0.05.

**Figure 3:**
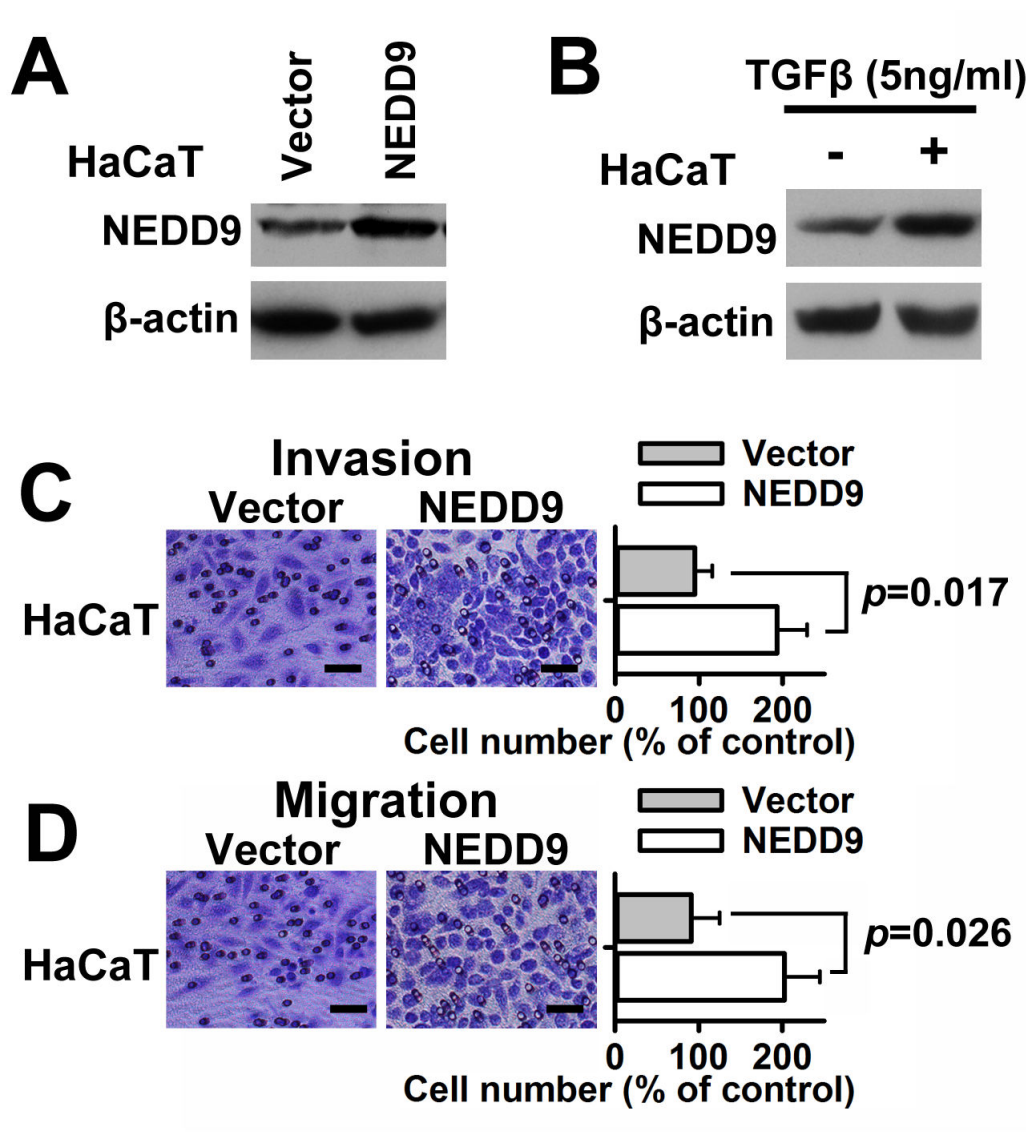
Overexpression of NEDD9 led to increased cell migration and invasion. Lentivirus vectors were employed to overexpress NEDD9 in human HaCaT keratinocytes (A). TGFβ (5ng/ml) also increased the expression of NEDD9 in human HaCaT keratinocytes (B). Results of Transwell assay showed that lentiviral delivery of NEDD9 resulted in increased cell invasion (C) and migration (D) in HaCaT cells without TGFβ stimulation. Scale bar, 100 μm.

To identify NEDD9 function in cervical cancer cells, we knocked down NEDD9 in SiHa and HeLa cells using a specific siRNA (S: 5' GAG ACA CCA UCU ACC AAG U[dT] [dT] 3', AS: 5' ACU UGG UAG AUG GUG UCU C[dT] [dT] 3'), which was selected from three candidates by qPCR. As shown in Figure 2A, 2B and 2C, the siRNA showed strong interference effects against NEDD9 mRNA and protein in cervical cancer SiHa and HeLa cells. Further, we tried to decide if migration and invasion of cervical cancer cells would be attenuated by reduction of NEDD9 expression via lentivirus-carried shRNA. The results of transwell assays showed that NEDD9 shRNA cut down invasive and migratory capability of cervical cancer SiHa and HeLa cells (Figure 2D and 2E). Moreover, the results of wound-healing assays showed significantly slow wound closure in the NEDD9 shRNA-treated vs. control SiHa and HeLa cells at 24 h after scratch (Figure 2F, 2G and 2H). We further determined the effects of increased NEDD9 on cell migration and invasion. Lentivirus vectors were employed to overexpress NEDD9 in HaCaT cells (Figure 3A). TGFβ (5ng/ml) also increased the expression of NEDD9 in HaCaT cells (Figure 3B). The results of Transwell assay showed that exogenous overexpression of NEDD9 resulted in increased cell invasion and migration in HaCaT cells without TGFβ stimulation (Figure 3C and 3D).

### Mutual regulation between NEDD9 and FAK or SRC

**Figure 4:**
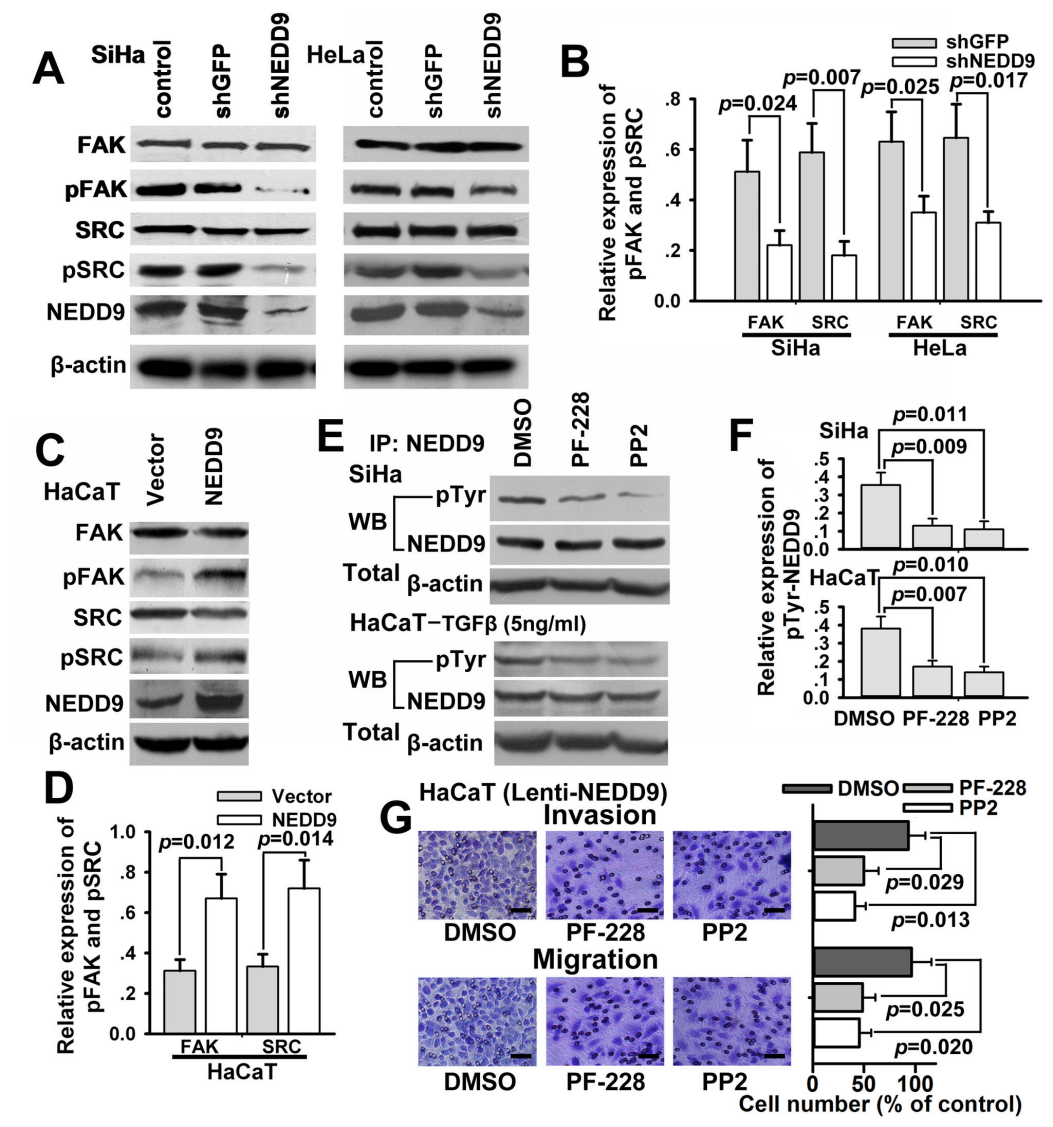
NEDD9 regulates tyrosine dephosphorylation of FAK and SRC. (A and B) Expression of NEDD9-associated oncoproteins, FAK and SRC, was examined by Western blotting. Tyrosine-phosphorylated FAK and SRC, rather than FAK and SRC, were significantly down-regulated while NEDD9 was silenced in SiHa and HeLa cells. (C and D) Moreover, tyrosine-phosphorylated FAK and SRC were significantly upregulated while NEDD9 was exogenously overexpressed in HaCaT cells. (E and F) Results of immunoprecipitation showed that tyrosine-phosphorylated NEDD9, rather than NEDD9, were significantly down-regulated while FAK or SRC was suppressed by FAK inhibitor PF-228 or SRC inhibitor PP2 in SiHa and TGFβ-stimulating HaCaT cells. (G) Results of Transwell assay showed that increased cell invasion and migration by exogenous overexpression of NEDD9 were suppressed by FAK inhibitor PF-228 or SRC inhibitor PP2 in HaCaT cells. Scale bar, 100 μm.

Furthermore, the expressions of FAK and SRC, two NEDD9-associated proteins [4], in infected SiHa and HeLa cells were determined by Western blot. The results showed that the expressions of phospho-FAK (Tyr397) and phospho-Src (Tyr416), but not total FAK and total SRC protein, were significantly decreased following knockdown of NEDD9 (Figure 4A and 4B). In overexpression experiments, tyrosine-phosphorylated FAK and SRC, rather than total FAK and total SRC, were significantly upregulated while NEDD9 was exogenously overexpressed in HaCaT cells (Figure 4C and 4D). Moreover, immunoprecipitation was employed to determine if FAK inhibitor PF-228 (Sigma, 10 μM) or SRC inhibitor PP2 (Sigma, 10 μM) regulated the expression of NEDD9. The results showed that the expression of tyrosine-phosphorylated NEDD9, rather than total NEDD9, was significantly decreased while FAK or SRC was suppressed by PF-228 or PP2 in SiHa and TGFβ-stimulating HaCaT cells (Figure 4E and 4F). Additionally, PF-228 (10 μM) or PP2 (10 μM) reduced phosphorylation of FAK or SRC in TGFβ-stimulating HaCaT cells (Figure S1). PF-228 or PP2 suppressed invasion and migration of SiHa and TGFβ-stimulating HaCaT cells (Figure S2). Similarly, the results of Transwell assay showed that both inhibitors suppressed cell invasion and migration increased by exogenous overexpression of NEDD9 in HaCaT cells (Figure 4G).

### NEDD9 regulates Vimentin and E-cadherin in cervical cancer cells

**Figure 5:**
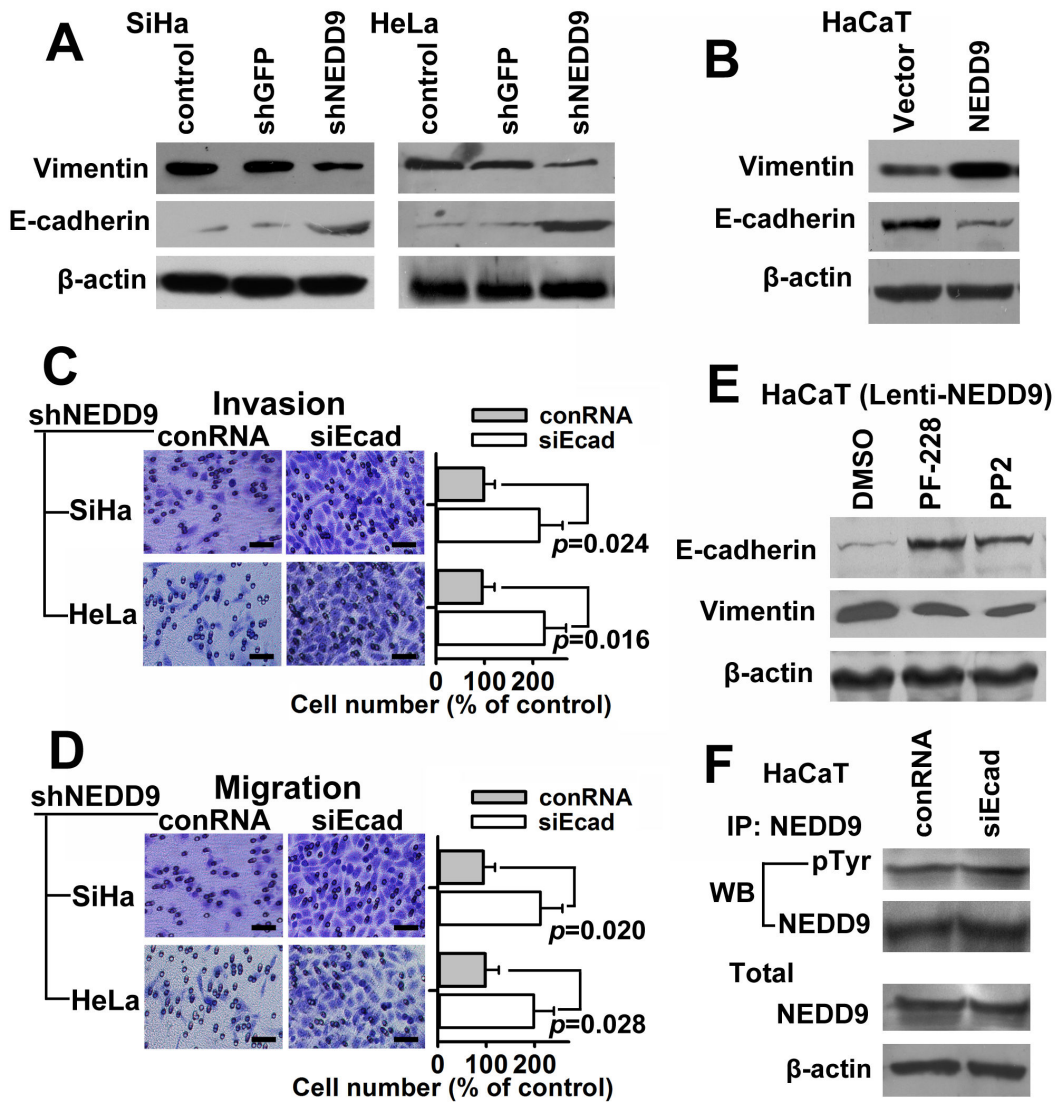
NEDD9 regulates Vimentin and E-cadherin expression. Expression of EMT markers, Vimentin and E-cadherin, was examined by Western blotting. (A) Vimentin was down-regulated and E-cadherin was upregulated while NEDD9 was silenced in SiHa and HeLa cells. (B) Vimentin was upregulated and E-cadherin was down-regulated while NEDD9 was overexpressed in HaCaT cells. Results of Transwell assay showed that decreased cell invasion (C) and migration (D) by shRNA of NEDD9 were increased by siRNA against E-cadherin in SiHa and HeLa cells. Scale bar, 100 μm. (E) E-cadherin was upregulated and Vimentin was down-regulated by virtue of FAK inhibitor PF-228 or SRC inhibitor PP2 in HaCaT cells with exogenous NEDD9. (F) Results of immunoprecipitation showed that tyrosine-phosphorylated NEDD9 and total NEDD9 were not significantly regulated while E-cadherin was suppressed by siRNA in HaCaT cells.

Vimentin and E-cadherin were important proteins in relation to cell invasion and migration. Western blot analysis showed that Vimentin was down-regulated and E-cadherin was upregulated while NEDD9 was suppressed by shRNA in SiHa and HeLa cells (Figure 5A). Moreover, Vimentin was upregulated and E-cadherin was down-regulated while NEDD9 was overexpressed in HaCaT cells (Figure 5B). The results suggest that Vimentin and E-cadherin are involved in the regulatory role of NEDD9 on cell migration and invasion in cervical cancer cells. The results of Transwell assay showed that decreased cell invasion and migration induced by shRNA specific for NEDD9 were increased by transfection of siRNA against E-cadherin (Figure 5C and 5D). The results further confirmed that E-cadherin was a suppressive regulatory protein in NEDD9 promoting cell migration and invasion in cervical cancer cells. Moreover, the down-regulated E-cadherin via overexpression of NEDD9 was significantly upregulated by virtue of PF-228 or PP2 in HaCaT cells, meanwhile, the up-regulated Vimentin expression was significantly down-regulated (Figure 5E). In the reverse experiment, neither tyrosine-phosphorylated nor total NEDD9 was significantly changed while E-cadherin was suppressed by siRNA in HaCaT cells (Figure 5F).

### E6/E7 oncoprotein does not regulate NEDD9 in cervical cancer cells

**Figure 6:**
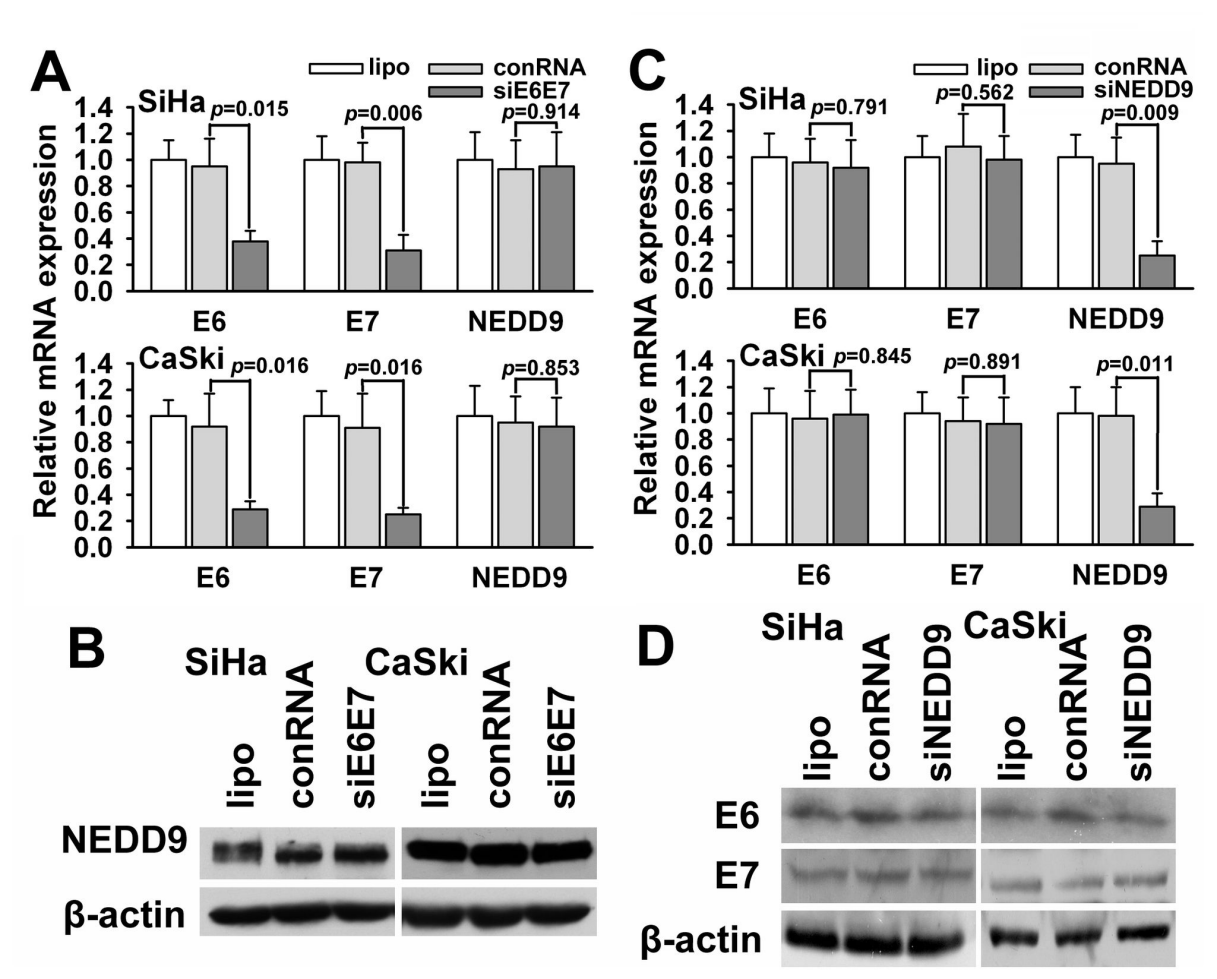
The association between NEDD9 and E6/E7 expression. (A and B) The result of qPCR and western blots showed that deprivation of E6/E7 by siRNA didn’t change the expression of NEDD9 in HPV-positive SiHa and CaSki cells. (C and D) Interference of NEDD9 didn’t affect the expression of E6/E7 in SiHa and CaSki cells.

It has been known that HPV E6 and E7 genes are key oncogenes in initiating the development of cervical cancer, but the effect of E6 and E7 on the host genes associated with the cancer progression remains uncertain. Thus, we observed possible links between E6/E7 and NEDD9 in cervical cancer cells. However, deprivation of HPV16 E6/E7 by siRNA didn’t change the expression of NEDD9 in HPV16-positive SiHa and CaSki cells (Figure 6A and 6B), vice versa, down-regulation of NEDD9 didn’t affect the expression of E6/E7 (Figure 6C and 6D). Our results suggest that NEDD9 is a late molecular event that participates mainly in the progression or metastasis of cervical cancer.

## Discussion

Although NEDD9 was initially first identified as one of new genes expressed by neural precursor cells but down-regulated following fetal development [3], several current studies found that NEDD9 overexpression, as oncogenic signaling abnormalities, was associated with cancer metastasis in breast cancer [6,20,21], glioblastoma [9], melanoma [10] and head and neck squamous cell carcinoma (HNSCC) [13], as well as linked to drug resistance in gastrointestinal stromal tumor (GIST) [22]. Recent studies showed that NEDD9 regulates TGFβ pathway in breast cancer [23,24] and hepatocellular cancer [25] and Wnt signaling in colorectal cancer [26]. Moreover, TGFβ is a potent inhibitor of cell cycle in human keratinocyte HaCaT cells, yet it has also been shown to promote invasion and migration in HaCaT cells [27,28]. Thus we employed HaCaT cells as a model to study an induced migration/invasion phenotype.

It had been proven by immunohistochemical analysis that NEDD9 was expressed in human bronchiolar epithelium [29], human lymphocytes in rheumatoid arthritis synovium [30], rat cerebral cortex and hippocampus neurons [31], human nevi and melanoma [10], murine embryonic encephalic and trunk neural tube [32], human late-onset Alzheimer’s disease [33], mouse and chick embryonic neural crest [34], human head and neck squamous cell carcinoma (HNSCC) [13]. Moreover, immunohistochemical analysis has also revealed that NEDD9 overexpression is correlated with HNSCC and melanoma progression [10,13]. Cervical cancer possesses a clinical characteristic that metastasis to lymph node usually occurs at early stage and presents poor prognosis. Thus, we detected and compared the NEDD9 expression by immunohistochemical analysis in 67 cervical cancer and 22 cervical normal tissues, as well as cervical cancer cell lines and non-tumor keratinocyte line. Our results revealed that NEDD9 protein was overexpressed in most cervical carcinoma tissues and cells compared with normal epithelium tissues and non-tumor keratinocytes. Further, we found that NEDD9 overexpression was correlated with lymph node metastasis, histological grading, and FIGO stage of cervical carcinoma patients. Our results were similar to those reported by Lucas JT, Jr. et al [13] in HNSCC and by Kim M et al [10] in melanoma and suggest that NEDD9 overexpression may involve in progression and metastasis of cervical cancer.

NEDD9 protein conserves a NH2-terminal Src homology 3 (SH3) domain, which binds to protein containing polyproline motifs such as FAK. FAK is one of the most important associated proteins of NEDD9 especially in the focal adhesion [4]. Natarajan M et al [9] thought that NEDD9 acted as specific downstream effectors of FAK that promoted glioblastoma cell migration and invasion. We found in the study that NEDD9 and FAK similarly distributed in cytoplasm of HPV16-positive cervical carcinoma SiHa cells (data not shown), suggesting there perhaps are some special links between NEDD9 and FAK. RNA interference (RNAi) is a powerful tool for silencing specific genes. Several studies [9,10,14,25,34-38] have shown that specific siRNAs targeting mRNA of NEDD9 gene are able to down-regulate expression of NEDD9. Here, we employed RNAi as a tool to investigate the role of NEDD9 in the progression of cervical cancer. Moreover, a lentivirus gene transfer vector was used to exogenously overexpress NEDD9 in the study. In our experiments, siRNAs targeting NEDD9 gene effectively inhibited both mRNA and protein expression of NEDD9. FAK and SRC were implicated as important targets of NEDD9 and they were phosphorylated and activated mutually, which was regarded as the “core regulation process” of NEDD9 [7]. Actually, functions of FAK and SRC proteins depend on their tyrosine phosphorylation in some specific amino acid residues in the proteins (for example, Tyr416 of SRC and Tyr397 of FAK). Thus, we detected tyrosine phosphorylation of FAK and SRC protein following down-regulation or upregulation of NEDD9 and found that silencing of NEDD9 resulted in tyrosine dephosphorylation, rather than decreased expression of both FAK and SRC. Interestingly, overexpression of NEDD9 led to tyrosine phosphorylation of FAK and SRC. Moreover, we also found tyrosine-phosphorylation rather than expression of NEDD9 was inhibited while the tyrosine-phosphorylation of FAK or SRC was suppressed by selective inhibitors such as PF-228 and PP2. Many groups have found NEDD9 is a downstream molecule of FAK and SRC which promote migration and invasion-related signaling [39,40]. However, several studies have recently shown that NEDD9 probably acted as upstream signal of FAK and SRC [6,41]. Persistently reduced FAK [6] and SRC [41] activation was shown in cells derived from NEDD9 knockout strain. Our findings suggest that there may be a positive feedback loop of tyrosine phosphorylation between NEDD9 and FAK or SRC. Hence, tyrosine phosphorylation was continuous strengthened via the so-called “core regulation process” and ultimately form signals of invasion and metastasis.

Since its initial functional analysis in 1996 [4,5], NEDD9 was associated with migration, invasion, and metastasis in different studies. Law et al. first discovered NEDD9 might regulate cell polarization [4], which was an indispensable step in the process of cell migration. Cell migration is the base of cancer cell invasion and metastasis. Up to now, NEDD9 was found to participate in cancer metastasis in glioblastoma [9], melanoma [10], and breast cancer. Currently, it is believed that NEDD9 protein is associated with migration, invasion and metastasis of cancer cells [7,42]. Our results showed that NEDD9 protein was overexpressed in cervical cancer tissues with metastasis. Moreover, inhibiting NEDD9 expression by RNAi attenuated migration and invasion of cervical cancer cells. Cells undergoing epithelial-mesenchymal transition (EMT) lose their epithelial morphology and acquire a motile phenotype, which plays a key role in the cancer invasion and metastasis [43]. Vimentin and E-cadherin were important biomarkers of epithelial-mesenchymal transition (EMT). Western blot analysis showed that shRNA against NEDD9 reduced expression of Vimentin and increased expression of E-cadherin and NEDD9 overexpression upregulated Vimentin and down-regulated E-cadherin, which is similar to those reported by Tikhmyanova N [21]. Furthermore, down-regulated E-cadherin via overexpression of NEDD9 was significantly upregulated by virtue of inhibitor of FAK or SRC. Neither tyrosine-phosphorylated nor total NEDD9 were significantly regulated while E-cadherin was suppressed. Our results suggest that NEDD9 overexpression promotes migration and invasion of cervical cancer cells probably via, at least partially, epithelial-mesenchymal transition (EMT) pathway. E-cadherin is a downstream protein of NEDD9 and may be a key modulator of NEDD9-mediated cancer cell migration and invasion.

It is well known that human papillomavirus (HPV) infection is the most important etiologic factor in cervical cancer [44]. Infection with high-risk HPV (hr-HPV), particularly HPV types 16 and 18, is causally linked to the development of cervical cancer. Numerous reports have established that high-risk HPV (hr-HPV) strains particularly HPV types 16 and 18 are oncogenic, which depends on the viral E6 and E7 oncogenes that target p53 and pRb tumor suppressor proteins [45]. Given the importance of E6/E7, we hypothesized that there were some links between E6/E7 and NEDD9 in the beginning of the study. However, we did not establish a connection between E6/E7 and NEDD9. Taking into account HPV infection is an early event in the development of cervical cancer, we believe that NEDD9 may be a late molecular event that participates mainly in the progression or metastasis of cervical cancer.

In summary, scaffolding protein NEDD9 is overexpressed in cervical cancer tissues and cells. Overexpressed NEDD9 promotes cell migration and invasion in cervical cancer cells, probably via regulating tyrosine dephosphorylation of FAK and SRC and the expression of EMT-associated protein E-cadherin. Our data implicate a positive feedback loop of tyrosine phosphorylation between NEDD9 and FAK or SRC, and E-cadherin may be a key molecule in NEDD9-mediated oncogenic signaling. These results suggest not only a mechanism for migration and invasion involving NEDD9-mediated oncogenic signaling but also novel therapeutic strategy and target for treating patients with advanced cervical cancer.

## Supporting Information

Table S1 **Clinicopathologic data of 89 cases in the study.**
(PDF)

Table S2 **Sequences for siRNAs, shRNA and the PCR primers used.**
(PDF)

Figure S1 **Reduced phosphorylation of FAK and SRC by PP-2 and PF-228 in TGFβ-stimulating HaCaT cells.** Expression of NEDD9-associated oncoproteins, FAK and SRC, was examined by Western blotting. Tyrosine-phosphorylated FAK (A and B) and SRC (C and D), rather than FAK and SRC, were significantly down-regulated by addition of PP-2 and PF-228 in TGFβ-stimulating HaCaT cells.
(TIF)

Figure S2 **Reduced cell invasion and migration by PP-2 and PF-228 in SiHa and TGFβ-stimulating HaCaT cells.** (A) Results of Transwell assay showed that increased cell invasion and migration by TGFβ stimulation were suppressed by FAK inhibitor PF-228 or SRC inhibitor PP2 in HaCaT cells. (B) Cell invasion and migration were suppressed by FAK inhibitor PF-228 or SRC inhibitor PP2 in SiHa cells. Scale bar, 100 μm.
(TIF)
