# Supplementary material for: The Overexpression of Scaffolding Protein NEDD9 Promotes Migration and Invasion in Cervical Cancer via Tyrosine Phosphorylated FAK and SRC
Source: PLoS One. 2013 Sep 18;8(9):e74594. doi: 10.1371/journal.pone.0074594 (PMC3776827; doi:10.1371/journal.pone.0074594)
Supplement: Table S1 — Clinicopathologic data of 89 cases in the study. (PDF) [file pone.0074594.s001.pdf]

Table S1. Clinicopathologic data of 89 cases in the study

Characteristic	SCC	AC	Normal
**n**	38	29	22
**Age**			
**Median**	46	41	50
**Range**	33-67	28-71	40-69
**Metastasis**			
**No-met**	22	22	
**Met**	16	7	
**FIGO stage**			
**I**	12	17	
**II**	8	4	
**III-IV**	18	8	
**Grade**			
**I**	8	7	
**II**	19	16	
**III**	11	6	

Abbreviations: SCC, squamous carcinoma of the cervix; AC, adenocarcinoma of the cervix.
