# Supplementary material for: The Overexpression of Scaffolding Protein NEDD9 Promotes Migration and Invasion in Cervical Cancer via Tyrosine Phosphorylated FAK and SRC
Source: PLoS One. 2013 Sep 18;8(9):e74594. doi: 10.1371/journal.pone.0074594 (PMC3776827; doi:10.1371/journal.pone.0074594)
Supplement: Table S2 — Sequences for siRNAs, shRNA and the PCR primers used. (PDF) [file pone.0074594.s002.pdf]

**Table S2. Sequences for siRNAs, shRNA and the PCR primers used**

| Name | Sequence (5' - 3') |
|---|---|
| siRNA1 against NEDD9 S | GAG ACA CCA UCU ACC AAG U[dT][dT] |
| siRNA1 against NEDD9 AS | ACU UGG UAG AUG GUG UCU C[dT][dT] |
| siRNA2 against NEDD9 S | UUA CAA AGC CCG UGG AGA A[dT][dT] |
| siRNA2 against NEDD9 AS | UUC UCC ACG GGC UUU GUA A[dT][dT] |
| siRNA3 against NEDD9 S | AGA CAC AGC UAU UGA GAG A[dT][dT] |
| siRNA3 against NEDD9 AS | UCU CUC AAU AGC UGU GUC U[dT][dT] |
| siRNA against E6/E7 S | GCA UGG AGA UAC ACC UAC A[dT][dT] |
| siRNA against E6/E7 AS | UGU AGG UGU AUC UCC AUG C[dT][dT] |
| siRNA against E-cadherin S | GGG UUA AGC ACA ACA GCA A[dT][dT] |
| siRNA against E-cadherin AS | UUG CUG UUG UGC UUA ACC C[dT][dT] |
| control siRNA S | UUC UCC GAA CGU GUC ACG U[dT][dT] |
| control siRNA AS | ACG UGA CAC GUU CGG AGA A[dT][dT] |
| shRNA against NEDD9 | GAG ACA CCA UCU ACC AAG U uucaagaga ACU UGG UAG AUG GUG UCU CUU |
| shRNA against GFP | GCU ACC UGU UCC AUG GCC A uucaagaga UGG CCA UGG AAC AGG UAG CUU |
| Primer NEDD9 S | ATG TCC ACG TCT TCC ACC TCC |
| Primer NEDD9 AS | AGT GAC CAG TGC CAT TAG GCT G |
| Primer E6 S | CTG CAA GCA ACA GTT ACT GC |
| Primer E6 AS | GGC TTT TGA CAG TTA ATA CAC C |
| Primer E7 S | CAT GGA GAT ACA CCT ACA TTG C |
| Primer E7 AS | CAC AAC CGA AGC GTA G AG TC |
| Primer GAPDH S | GAC AGT CAG CCG CAT CTT CT |
| Primer GAPDH AS | TTA AAA GCA GCC CTG GTG AC |
