# Supplementary figures and images for: The Overexpression of Scaffolding Protein NEDD9 Promotes Migration and Invasion in Cervical Cancer via Tyrosine Phosphorylated FAK and SRC
Source: PLoS One. 2013 Sep 18;8(9):e74594. doi: 10.1371/journal.pone.0074594 (PMC3776827; doi:10.1371/journal.pone.0074594)

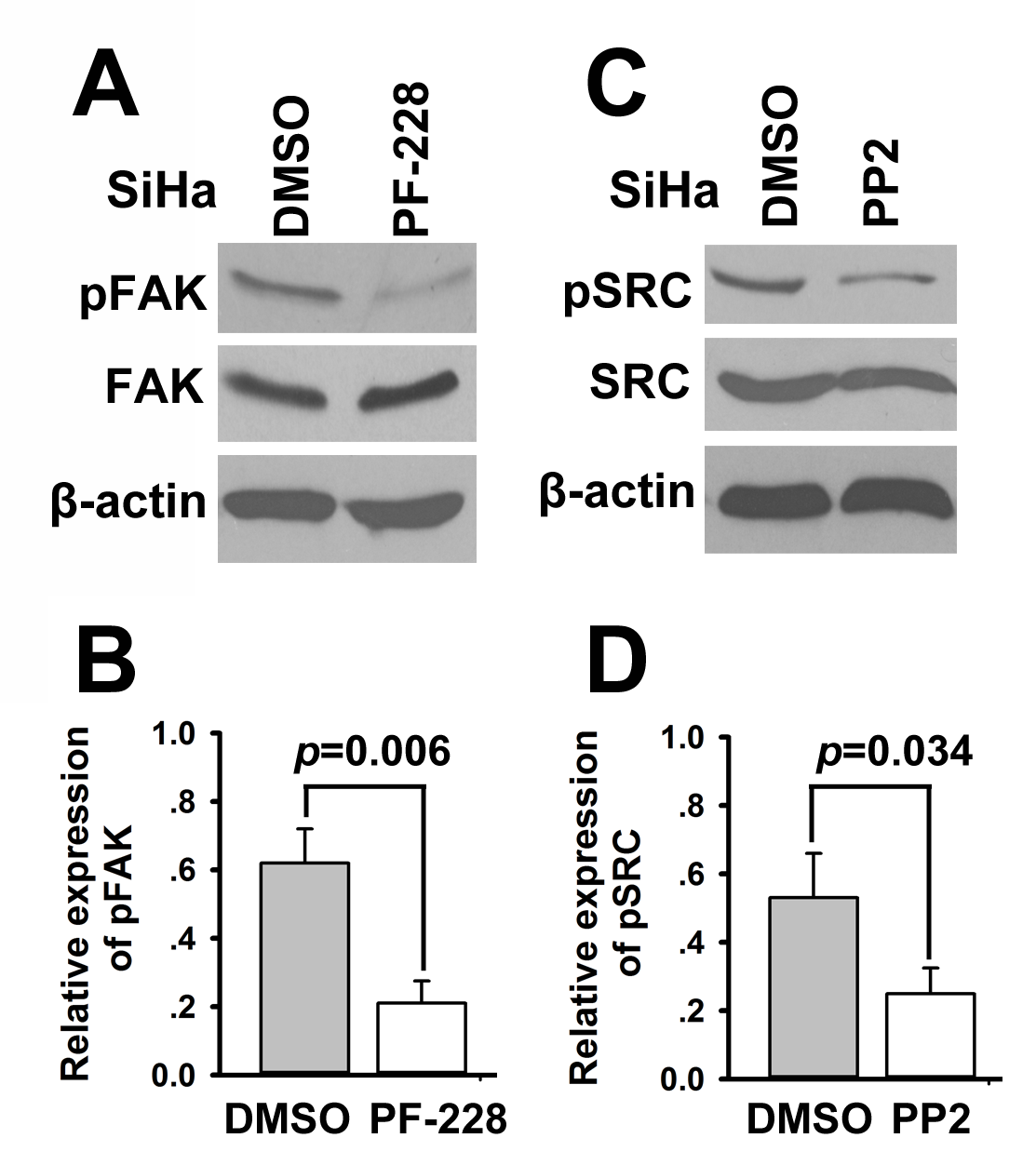

Supplement: Figure S1 — Reduced phosphorylation of FAK and SRC by PP-2 and PF-228 in TGFβ-stimulating HaCaT cells. Expression of NEDD9-associated oncoproteins, FAK and SRC, was examined by Western blotting. Tyrosine-phosphorylated FAK (A and B) and SRC (C and D), rather than FAK and SRC, were significantly down-regulated by addition of PP-2 and PF-228 in TGFβ-stimulating HaCaT cells. (TIF) [file pone.0074594.s003.tif]

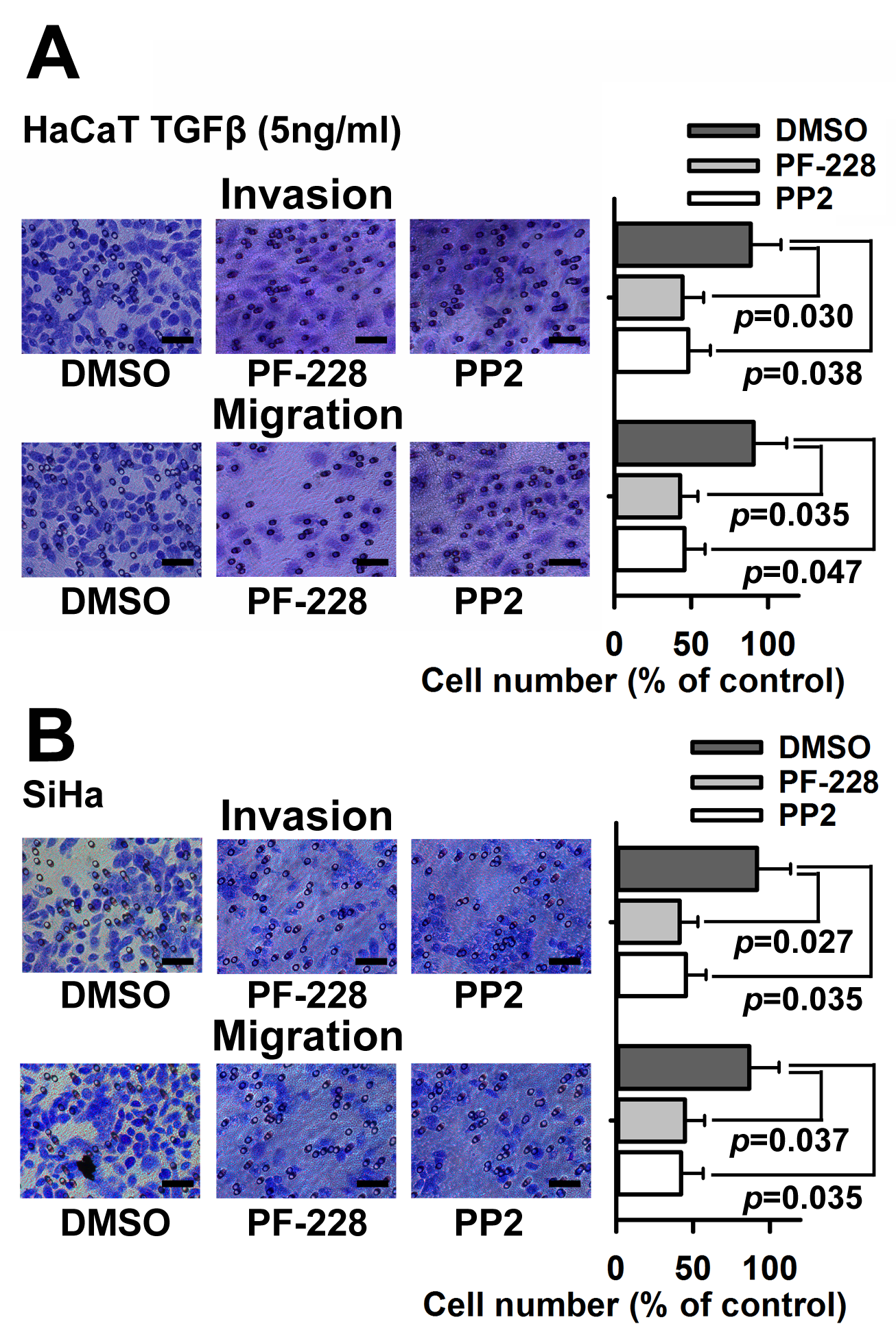

Supplement: Figure S2 — Reduced cell invasion and migration by PP-2 and PF-228 in SiHa and TGFβ-stimulating HaCaT cells. (A) Results of Transwell assay showed that increased cell invasion and migration by TGFβ stimulation were suppressed by FAK inhibitor PF-228 or SRC inhibitor PP2 in HaCaT cells. (B) Cell invasion and migration were suppressed by FAK inhibitor PF-228 or SRC inhibitor PP2 in SiHa cells. Scale bar, 100 μm. (TIF) [file pone.0074594.s004.tif]
